# Supplementary material for: Vitamin D Modified DSS-Induced Colitis in Mice via STING Signaling Pathway
Source: Biology (Basel). 2025 Jun 18;14(6):715. doi: 10.3390/biology14060715 (PMC12190092; doi:10.3390/biology14060715)
Supplement: Supplementary file 1 [file biology-14-00715-s001.zip › Supplementary Materials S1: 16s-report of gut microbiota/Groups/alphadiv/Alpha_diversity_group.pdf]

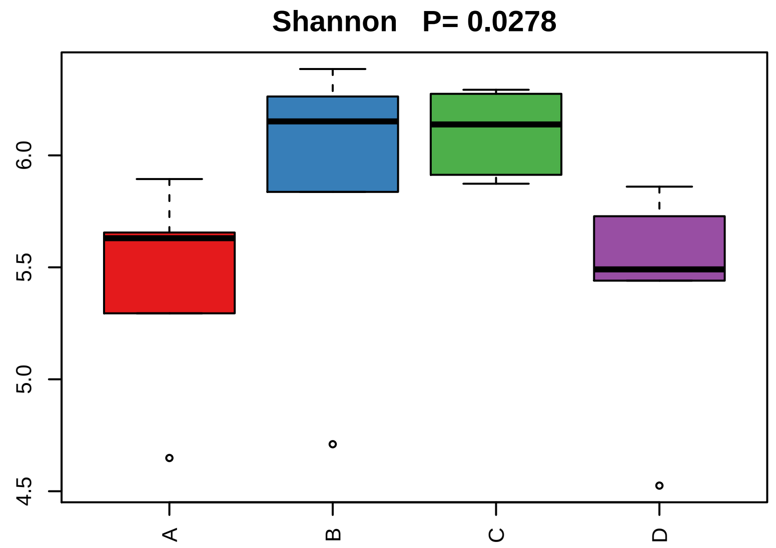

Shannon P= 0.0278
6.0
5.5
5.0
4.5
A
B
C
D

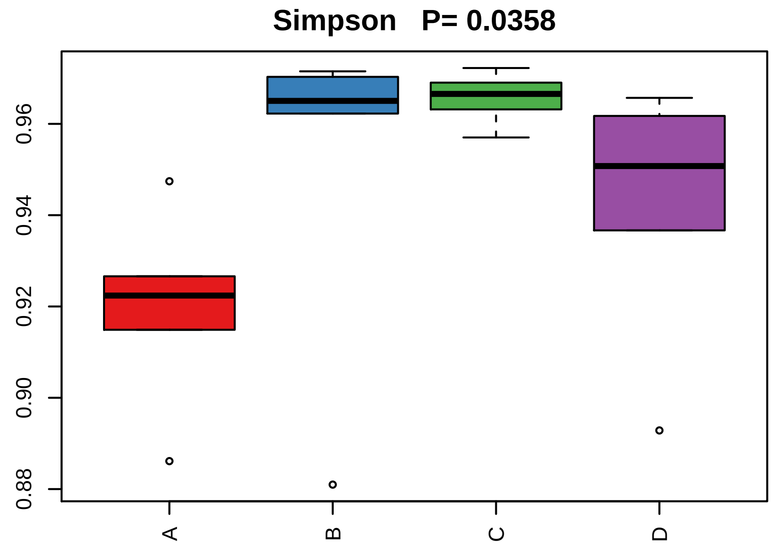

Simpson P= 0.0358
0.96
0.94
0.92
0.90
0.88
A
B
C
D

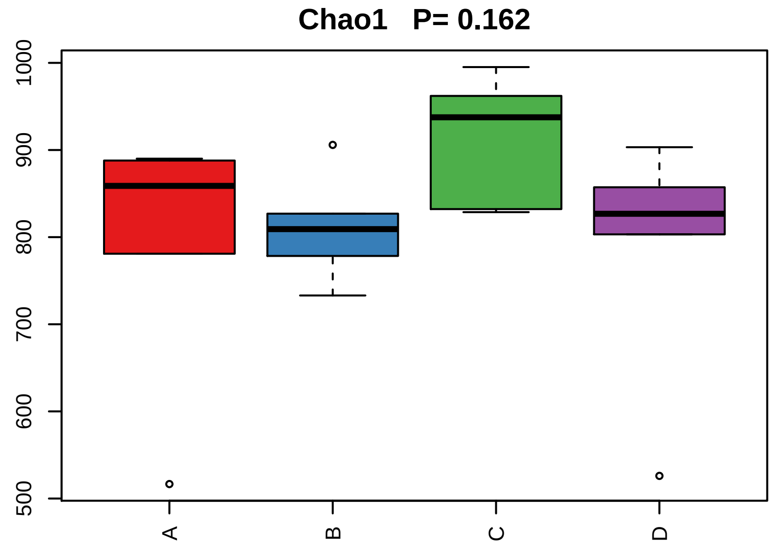

Chao1 P= 0.162
1000
900
800
700
600
500
A
B
C
D
